# Different dry-wet pulses favor different functional strategies: A test using tropical dry forest tree species

**DOI:** 10.1371/journal.pone.0309510

**Published:** 2024-12-03

**Authors:** Flor Vega-Ramos, Lucas Cifuentes, Fernando Pineda-García, Todd Dawson, Horacio Paz

**Affiliations:** 1 Instituto de Investigaciones en Ecosistemas y Sustentabilidad, Universidad Nacional Autónoma de México, Morelia, Michoacán, México; 2 Departamento de Ciencias Forestales, Sede Medellín, Facultad de Ciencias Agrarias, Universidad Nacional de Colombia, Medellín, Colombia; 3 Escuela Nacional de Estudios Superiores, Unidad Morelia, Universidad Nacional Autónoma de México, Morelia, Michoacán, México; 4 Center for Stable Isotope Biogeochemistry and the Department of Integrative Biology, University of California, Berkeley, CA, United States of America; Qingdao Agricultural University, CHINA

## Abstract

In many terrestrial habitats, plants experience temporal heterogeneity in water availability both at the intra and inter annual scales, creating dry-wet pulse scenarios. This variability imposes two concomitant challenges for plants: surviving droughts and efficiently utilizing water when it becomes available, whose responses are closely interconnected. To date, most studies have focused on the response to drought following static designs that do not consider consequences of repeated transitions from one state to the other. In principle, different dry-wet pulse scenarios among years may differentially affect species performance, plant strategies, and promote coexistence through temporal niche separation. We predicted that short frequent droughts would disfavor drought-avoidant species, as rapid leaf loss and production could disrupt their carbon balance, whereas tolerant species, which maintain carbon gain during droughts, should thrive in such conditions. Prolonged droughts might harm tolerant species by causing severe cavitation. We assessed the survival and growth responses of seedlings from 19 tropical dry forest tree species to simulated natural dry-wet pulse scenarios, examining their relationships with the continuum of species’ functional strategies under field conditions, and used greenhouse experiments to accompany the field experiment. As expected, different dry-wet pulse scenarios favored different plant functional strategies. Contrary to predictions, the most tolerant outperformed the most avoiders under all drought scenarios, while rapid water-exploiters thrived under non-drought conditions. The superiority of tolerant over avoider species was reverted in the greenhouse, suggesting that in addition to physiology, the fate of species may depend on extrinsic factors as natural enemies. The interplay between the marked variability of dry-wet pulse scenarios across the years and the diversity of water use strategies may contribute to species coexistence in the tropical dry forests. This research is relevant in predicting changes in dominant tree species under future climate scenarios characterized by increased temporal variation in water availability.

## Introduction

Terrestrial ecosystems are temporally heterogeneous in precipitation and thus water availability, which has been widely recognized to control ecosystem processes and species distribution, particularly in dry, semiarid and seasonal biomes [1, 2]. Temporal heterogeneity generates alternating pulses of water availability and shortage that differ strongly in duration and frequency among years and communities, generating inter-annual variation in the length of the dry- and rainy seasons as well as intra-annual variation in dry spells [2–4]. An overarching hypothesis in ecology predicts that under scenarios of high temporal heterogeneity, plants have evolved along a trade-off between the ability to tolerate the resource shortage and survive, and the ability to efficiently acquire the resources when they are available, maximizing growth and thus competitive advantage [1, 5, 6]. One prediction is that some species will specialize to resist drought while others avoid drought and exploit water along a continuum of strategies, which may contribute to species coexistence within the same community [7–9]. This hypothesis is typically tested by comparing performance and functional traits among species when subjected to single drought and no water limitation treatments, where the interruption of drought by rewatering is solely used to verify final survival [e.g., 10–13], In other words, most of studies do not aim to understand the consequences of the occurrence of repeated dry-wet pulses [14, 15]. The legacies of previous droughts as well as cumulative effects of repeated drought events are commonly discussed in the literature but few studies evaluate explicitly such effects [16]. This is a relevant limitation, since many seasonally dry regions on Earth undergo erratic dry-wet pulses even during the rainy season, exposing plants to the potential for physiological shock from the repeated sudden transitions between full growth/water use and water limitation/hydric stress [2, 4, 10, 15, 17].

Different pulse scenarios could select for different plant strategies, thus promoting species coexistence. For example, short and frequent dry pulses could select against species that avoid drought by readily shedding their leaves since the rapid loss and production of tissues may disrupt individuals’ carbon balance [12, 17]. Meanwhile long and infrequent pulses my select against non-deciduous species that keep working even at high levels of dehydration because they are at high risk of reaching thresholds of massive hydraulic failure [13]. Thus, asking what sets of functional traits enable species to deal with dry-wet pulses of differing lengths and frequencies is a relevant question that remains poorly explored. Given that climate change is expected to increase temporal heterogeneity of rainfall [18] understanding how functional traits relate to species’ performance under pulse-driven scenarios is critical for predicting changes in species coexistence and diversity and managing species and forests [17, 19].

The tropical dry forest is an ideal system to assess these questions, since these communities are subject to erratic variation in precipitation among and within years, and dry spells within the rainy season cause high mortality of young plants [10, 20]. In tropical dry forests, dry spells can last from a few days to months [11, 21, 22] creating a variety of dry-wet pulse scenarios that differ in frequency and duration. There is also well-documented continuum variation in trees’ functional strategies for using water and confronting drought. The most drought-tolerant species have high wood density with high xylem cavitation resistance and are capable to sustain low rates of photosynthesis long during the drought periods [13, 23]. In contrast, fast growing species have large leaves, high specific leaf area, low wood density and low xylem cavitation resistance; they are very sensitive to drought but have high water exploitation capacity and exhibit high growth rates under conditions of water availability [7, 24]. Other species with drought sensitive xylem avoid drought by shedding leaves quickly during drought and by using water and carbohydrate storages to buffer physiological balances [13, 25]. These plants pay respiratory costs while dormant, and produce new leaves when water becomes available yielding higher growth rates than tolerant but lower than fast growing species [13]. Previous studies have reported ample interspecific variation in wilting progression, damage of stems by massive cavitation (dieback), as well as survival after progressive droughts in seedlings [11]. The wide continuous variation of wood, leaf and drought phenology detected among dry tropical forest species [26] makes this system ideal to explore the impact of functional traits on plant responses to dry-water pulse scenarios.

In this study, we explored the hypothesis that plant species’ success depends not only on the dry-wet pulse scenario to which they are subjected, but also on the plant’s strategy, as driven by the species’ functional traits. We addressed two questions: (1) How do different drought frequency–duration scenarios (dry-wet pulse scenarios) impact growth and survival in tropical dry forest tree species? (2) Do different dry-wet pulse scenarios favor different plant strategies? We predicted that short, frequent droughts would act against readily deciduous drought-avoidant species because the repeated loss and production of leaves will lead to carbon unbalance, while favor the most tolerant species that simply retain their leaves while maintaining a low risk of hydraulic failure. On the contrary, we predicted that long droughts favor species that rapidly avoid drought by shedding leaves and act against the most tolerant species that eventually suffer massive hydraulic failure. Fast-growing species with high capacity for water use might be favored in scenarios with no drought, where they realize the highest growth rates.

To address these questions, we exposed seedlings of 19 common tropical dry forest tree species to several cycles of dry-wet pulse scenarios that are frequently observed in the study region. We measured functional traits to characterize the interspecific continuum of water use strategies and search for the effects of specie´s strategy on plant performance and the way they change among the dry-wet pulse scenarios. These interactions were assessed in a field experiment and accompanied by key greenhouse experiments to gain a better understanding of the effects while controlling by natural enemies.

## Methods

### Study site and species

The study was conducted at the Chamela-Cuixmala Biosphere Reserve on the Pacific coast of Mexico (19°30’ N, 105°03’ W). The main vegetation type is Tropical Dry Forest developing between 50 and 250 m.a.s.l., characterized by old growth trees with a canopy height of 7–15m [27]. The mean annual temperature is 25.6°C and the mean annual rainfall is 800.4 mm but is highly variable among years (340–1329 mm) with a marked dry season from November to May. There is high intra-annual variation in rainfall, leading to pulses of water availability and water shortage that vary in frequency and duration [27]. In dry years (< 400 mm pp), repetitive long-lasting droughts (15–31 days long with soil water potential < 5 MPa) occur during the rainy season, while in wet years (> 900 mm pp), short droughts (5–10 days long with soil water potential < 5 MPa) are frequent [22].

To assure a wide variation of interspecific functional strategies in our study, we selected 19 common tree species spanning a wide range in wood density and leaf phenology (Table 1) key traits considered as gross proxies of species water use and drought resistance strategies [28]. We harvested seeds from wild trees of each species during its peak fruiting period. The seeds were then germinated and grown in a greenhouse for three months prior to the experiment.

**Table 1:** List of the study species with their abbreviation.

Acronym	Species	Family
Acfa ^1^^,^^2^	*Acacia farnesiana* (L.) Willd.	Fabaceae
Amad ^1^^,^^3^	*Amphipterygium adstringens* Schltdl	Anacardiaceae
Appa ^1^^,^^2^	*Apoplanesia paniculata* C. Presl	Fabaceae
Caer ^1^^,^^2^^,^^3^	*Caesalpinia eriostachys* Benth	Fabaceae
Capl ^1^^,^^2^^,^^3^	*Caesalpinia platyloba* S. Watson	Fabaceae
Ceae ^1^^,^^2^^,^^3^	*Ceiba aesculifolia* (Kunth) Britten & Baker f.	Malvaceae
Coal ^1^^,^^2^^,^^3^	*Cordia alliodora* (Ruiz & Pav.) Oken	Cordiaceae
Coel ^1^^,^^2^^,^^3^	*Cordia elaeagnoides* DC.	Cordiaceae
Cral ^1^^,^^2^^,^^3^	*Crescentia alata* Kunth	Bignoniaceae
Ency ^1^^,^^2^^,^^3^	*Enterolobium cyclocarpum* (Jacq.) Griseb.	Fabaceae
Glse ^1^^,^^2^^,^^3^	*Gliricidia sepium* (Jacq.) Kunth ex Walp.	Fabaceae
Guul ^1^^,^^2^^,^^3^	*Guazuma ulmifolia* Lam.	Malvaceae
Ipwo ^1^^,^^2^^,^^3^	*Ipomoea wolcottiana* Rose	Convolvulaceae
Miar ^1^	*Mimosa arenosa* (Willd.) Poir.	Fabaceae
Pico ^1^^,^^2^^,^^3^	*Piptadenia constricta* (Micheli & Rose ex Micheli) J.F.M	Fabaceae
Pidu ^1^^,^^2^^,^^3^	*Pithecellobium dulce (Roxb*.*) Benth*.	Fabaceae
Rupa ^1^^,^^2^^,^^3^	*Ruprechtia pallida* Standl.	Polygonaceae
Sppu ^1^	*Spondias purpurea* L.	Anacardiaceae
Swhu ^1^^,^^2^^,^^3^	*Swietenia humilis* Zucc.	Meliaceae

^1^ indicates species included in the dry-wet pulses experiment done in a common garden

^2^ species included in the dry-wet pulses experiment done in greenhouse conditions

^3^ species included in the prolonged drought greenhouse experiment to provoke dieback. Species names according to www.tropicos.org.

#### Plant functional traits measurements

To characterize specie´s functional strategies before the onset of the dry-wet pulse experiments, we harvested ten, 3-months-old seedlings per species and measured 13 functional traits involved in plant strategies to use water and deal with drought (Table 2). Mean values per species were obtained for: leaf dry mass content (LDMC), specific leaf area (SLA), minimum photosynthetic unit size (MPS), minimum leaf water potential (Ψ_min_), wood density (WD), stem water content (SWC), bark water content (BWC), specific root length (SRL), root water content (RWC), vertical root elongation rate (VRER), total root biomass: leaf biomass ratio (RB/LB), fine root biomass: leaf biomass ratio (FRB/LB), fine root length: total leaf area ratio (FRL/LA), by following standard procedures (S1 Text).

**Table 2:** List of functional traits measured for 19 tropical dry forest species and their functional role as reported elsewhere.

Trait	Abbreviation and units	Level	Functional role	Description/significance and references
Minimum photosynthetic unit size	MPS (cm^2^)	Leaf	Drought tolerance	Indicator of the transpiration area and potential leaf cooling [29, 30]
Leaf dry matter content	LDMC (%)	Leaf	Drought tolerance	Indicator of leaf hydraulic safety and drought tolerance, leaf cost and herbivory defense [31, 32]
Minimum leaf water potential	Ψ_min_ (MPa)	leaf	Drought tolerance	Indicator of drought tolerance; maximum water stress level while maintaining minimal photosynthesis [33, 34]
Leaf retention time	LRT (days)	leaf	Drought avoidance	Indicator of speed of leaf area reduction in response to drought; drought avoidance [7, 35]
Specific leaf area	SLA (cm^2^·g^-1^)	Leaf	Water exploitation	Indicator of light capture efficiency per gram of leaf mass invested and photosynthetic rate [36]
Wood density	WD (cm^3^·g^-1^)	Stem	Drought tolerance	Indicator of xylem hydraulic conductivity and embolism resistance [37, 38]
Stem water content	SWC (%)	Stem	Drought avoidance	Indicator of water storage capacity of the stem tissues including bark, xylem and parenchyma; amount of water that can be released for a given change in water potential of the stem tissues; medium-term sustainable water reserves for transpiring leaves [39, 40]
Bark water content	BWC (%)	Stem	Drought avoidance	Indicator of stored water potentially used for maintaining xylem function; ability to mobilize the phloem elements efficiently [41, 42]
Specific root length	SRL (cm·g^-1^)	Root	Water exploitation	Indicator of the exploitation and foraging capacity of soil volume for water capture per unit biomass invested in the fine roots; characterizes the economic aspects of the root system [43]
Root water content	RWC (%)	Root	Drought avoidance	Indicator of water storage capacity of the root tissues. Water reserves with little exposure to desiccation that can be released for a given change in water potential of the root tissues [28, 39]
Vertical root elongation rate	VRER (cm·day^-1^)	Root	Water exploitation	Indicator of the capacity to forage for water deep in the soil [44, 45]
Total root biomass/total leaf biomass	RB/LB (g·g^-1^)	Whole plant	Water exploitation	Carbon allocation to above- versus below-ground organs [44]
Fine root biomass/total leaf biomass	FRB/LB (g·g^-1^)	Whole plant	Water exploitation	Mass based indicator of the water supply to foliar tissue [44]
Fine root length/ total leaf area	FRL/LA (cm·cm^-2^)	Whole plant	Water exploitation	Surface based indicator of the water supply to foliar tissue [44]

#### Field common garden experiment

A common garden watering experiment was established to expose seedlings of each species to simulated dry-wet pulse scenarios previously observed in the study area [22]. The 608 m^2^ (20 m x 35 m) experimental plot was established in a flat area at the Chamela Biological Station. This area was cleared of vegetation, fenced, and covered with clear plastic sheeting and 50% shade cloth in a v-shaped roof 4 m tall to divert rainwater. At the ground level, to divert rainwater out from the plot, we excavated 30 cm deep trenches with a 5% inclination and covered with plastic sheeting. A 2 m-wide edge corridor was left around the periphery of the plot, and the remaining area was subdivided into 20, 4m x 1m subplots to which the four watering treatments were assigned randomly. To avoid humidity contamination between adjacent sub-plots, we installed a plastic sheet barrier up to 50 cm depth along the perimeter of each sub-plot and left 2 m-wide corridors. In each sub-plot, 12 seedlings of each species were randomly planted. Sixty days after planting, four treatments simulating different scenarios of dry-wet pulses were applied by manually watering as follows: i) short and frequent drought pulses (SFD), consisting of ten days of watering then 15 days with no watering; seven cycles were simulated over 175 days; ii) long and infrequent drought pulses (LID), consisting of ten days of watering then 30 days with no watering; four cycles were simulated over 180 days; iii) prolonged droughts (PD) consisted of no watering until all individuals lost 100% of their leaves, which occurred after 175 days; and iv) no-drought (ND), consisting of continuous watering, with no drought pulses. Each simulated rainfall (wet pulse) during the experiment consisted of applying the equivalent of 20 mm of rainfall every two days. The control, SFD and LID included 17 species, while the PD treatment included two extra species (*Spondias purpurea*, *Mimosa arenosa*). Because several plants did not survive after the transplant, the number of individuals per species per subplot entering to the experiment varied between 4 and 12, yielding a total per species per treatment between 20 and 60. The diameter at the base and height of all seedlings were measured both at the beginning and the end of the experiment to estimate their relative growth rate (RGR). At the end of the experiment, all seedlings were watered for one month to determine survival. Air temperature and humidity and the soil water potential at different depths were monitored throughout the experiment in all treatments (S2 Text).

#### Greenhouse experiments

To better understand the effects of drought pulses on plant performance based on their functional traits, while controlling for other environmental factors, we performed two greenhouse experiments in Chamela. First, we simulated one short frequent dry-wet pulse, a scenario we expected to disfavor drought-avoidant species. Secondly, we simulated a prolonged drought (expected to disfavor drought-tolerant species) and evaluated how quickly individuals suffered massive damage from cavitation and their ability to survive it.

Seedlings of a subset of 16 species were grown in individual pots (20 cm diameter x 40 cm depth) containing 50:50 forest soil and riverbank soil until they reached four months of age. Half of the seedlings were subjected to six dry-wet pulses consisting of 10 days of watering to saturation, followed by 15 days with no watering, and the other half to a no-drought treatment as a control. According to plants availability, 17 to 25 seedlings of each species were assigned to 10 blocks representing the dry-wet pulse treatment, while 15 to 27 were assigned to 10 blocks representing the control. Atmospheric conditions and soil water potential were continuously monitored in 7 to 9 pots per species per treatment (S2 Text).

In addition, seedlings from a subset of 15 species were subjected to a progressive drought experiment under greenhouse conditions to quantify sensitivity to drought damage (dieback). Twenty seedlings of each species were grown under no water limitation in pots as described above until reaching four months. Then, seedlings were exposed to progressive desiccation by stopping watering. The wilting condition of each sapling was determined twice a week for the first 3 months, then once monthly thereafter. Previous desiccation trials in the same greenhouse allowed us to characterize and recognize dieback damage to the main stem in each species, which we assumed to indicate massive cavitation. When each plant presented evidence of 30% dieback of its main stem, the plant was immediately rewatered continuously to record survival one month later. We therefore had two parameters per species: 1) the mean number of days to reach 30% dieback (hereafter called vulnerability to cavitation damage), and 2) the likelihood of dying from intense drought.

### Data analysis

To understand patterns of trait covariation among species and define continuous axes describing plant strategies, we performed PCA based on simple mean values per species, which were log-transformed to meet normality and homogeneity assumptions. Correction for potential plant size effects were unnecessary as no significant trait-size regressions were detected (data not shown). The two first multivariate axes (PC1, PC2) were used as descriptors of the continuum of plant functional strategies.

To evaluate the effects of the dry-wet pulse scenarios on seedling´s survival and RGR, we performed linear mixed models with the species as a random factor and contrasted pairs of dry-wet pulse scenarios using lme4 [46] and the emmeans [47] in the R language version 4.2.2 [48].

To address our main question; how the dry-wet pulse scenario shapes the relative success of functional strategies of trees, we modelled survival and RGR as a function of plant functional strategy and looked for the interaction with the dry-wet pulse scenario. To do so we performed GLM mixed models using species’ scores on PC1 or PC2 as a regressor, the dry-wet pulse scenario as a fixed factor, and the species as a random factor. In these models, to control for potential effects of initial plant size, we included individual plant height as a covariate, as follows:


$$Survival∼height+PC(i)+(P{C(i))}^{2}+Treatment+PCA(i)+Treatment+(P{C(i))}^{2}\times Treatment+(Species)+\varepsilon$$ (1)

$$RGR∼height+PC(i)+(P{C(i))}^{2}+Treatment+PCA(i)\times Treatment+(P{C(i))}^{2}\times Treatment+(Species)+\varepsilon$$ (2)

Where (i) is the first or second principal component. We analyzed survival using binomial generalized linear mixed models, while RGR using linear mixed models. Because we expected quadratic responses of RGR and survival along the axes defining plant strategies, we first tried full quadratic models, and then linear models, retaining the terms height, treatment, PC(i) and the random (species) in the minimal model. Given the markedly uneven proportion of surviving individuals “1” values or dead individuals “0” values in certain dry-wet pulse scenarios, we used the complementary clog-log link function (instead of the simple logit function), to allow for asymmetrical survival responses along the covariate, following [49]. All mixed models were performed using the glmer (for survival) and lmer (for RGR) functions in lme4 [48]. To assess the fit of the linear vs. quadratic models, we used the marginal and conditional R^2^ estimators [50], using the r.squared GLMM function in the MuMIn package [51]. When the analysis of deviance detected a significant interaction between the dry-wet pulse scenario and the covariate, we compared the slopes between all pairs of dry-wet pulse scenarios using Tukey’s test. In the case of RGR, because quadratic trends were detected for both PC1 and PC2 regressors, the behavior of trends were compared among treatments by testing for differences between predicted means at low (-0.5), intermediate (0.0, 0.5) and large values (3.5, 2.0) of the regressor (PC1 and PC2 scores, respectively), by using the emmeans function in the multcomp package.

To evaluate the interaction between plant functional strategies and dry-wet pulse scenario on survival under greenhouse conditions, we used a similar modelling as that described for the field experiment. In this case only two scenarios were considered: short dry-wet pulses and no drought. Finally, to assess the effects of plant functional strategies on survival under a prolonged drought simulated in the greenhouse, we also applied a GLMM.

## Results

### Plant functional dimensions and strategies

**Fig 1:**
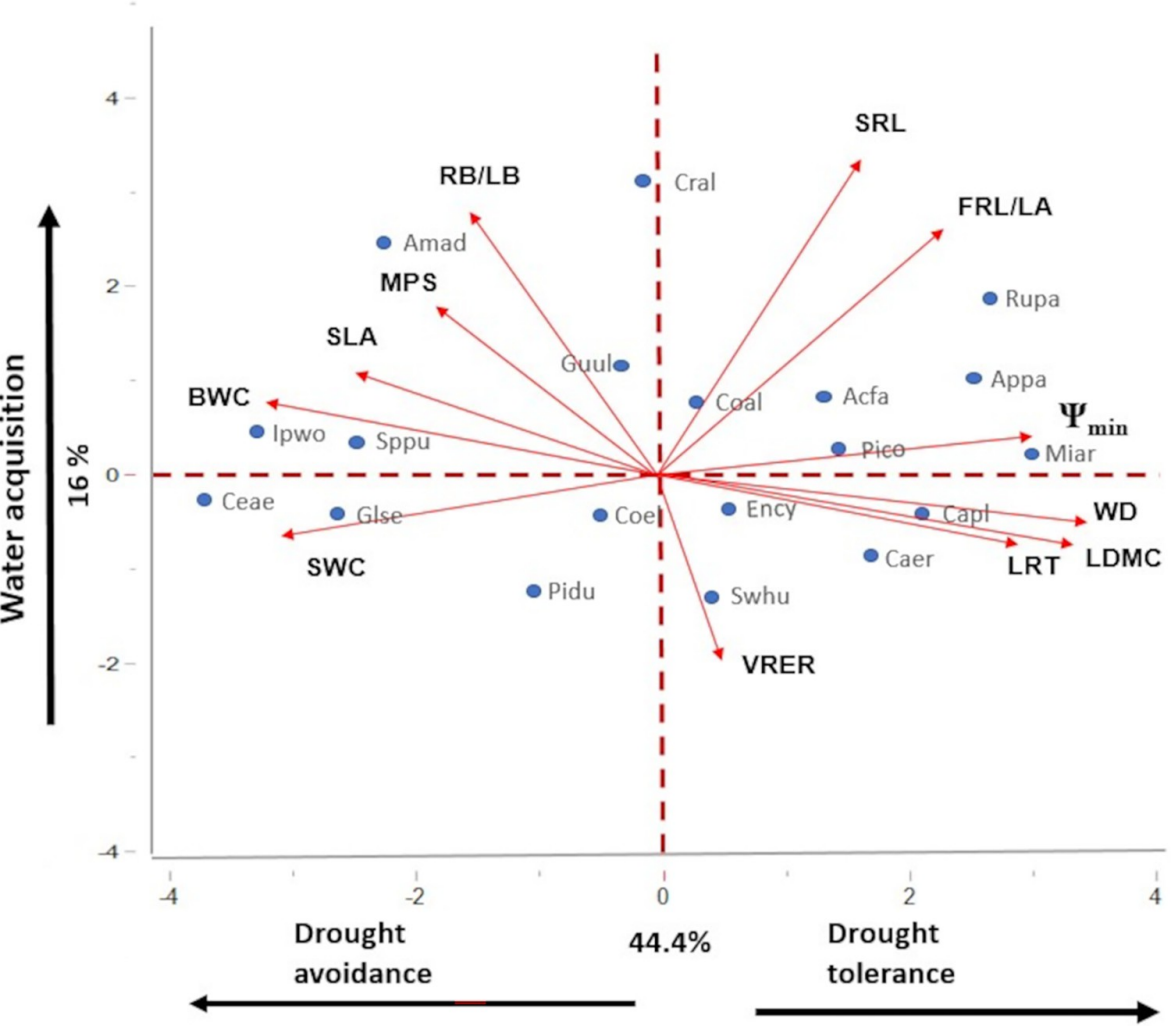
Principal component analysis of 12 traits in 19 tree species from the tropical dry forest, evaluated in seedlings. Species and trait abbreviations are the same as in Tables 1 and 2, respectively. Traits: MPS: Minimum photosynthetic unit size, LDMC: Leaf dry matter content, Ψ_min_: Minimum leaf water potential, LRT: Leaf retention time, SLA: Specific leaf area, WD: Wood density, SWC: Stem water content, BWC: Bark water content, SRL: Specific root length, RWC: Root water content, VRER: Vertical root elongation rate, RB/LB: Total root biomass/total leaf biomass, FRL/LA: Fine root length/ total leaf area. Species: Acfa: *Acacia farnesiana*.; Amad: *Amphipterygium adstringens*; Appa: *Apoplanesia paniculatta*; Caer: *Caesalpinia eriostachys*; Capl: *Caesalpinia platyloba;* Ceae: *Ceiba aesculifolia*; Coal: *Cordia alliodora*; Coel: *Cordia elaeagnoides*; Cral: *Crescentia alata;* Ency: *Enterolobium cyclocarpum*; Glse: *Gliricidia sepium*; Guul: *Guazuma ulmifolia; Ipwo*: *Ipomoea wolcottiana;* Miar: *Mimosa arenosa*; Pico: *Piptadenia constricta*; Pidu:*Pithecellobium dulce;* Rupa: *Ruprechtia pallida;* Sppu: *Spondias purpurea;* Swhu: *Swietennia hummillis*.

The traits multivariate analysis exhibited a strong covariation among most of measured traits along the first principal component (Fig 1 and S1 Table), which explained 44.4% of total variation. Positive PC1 scores denoted species with low (i.e., more negative) values of Ψ_min_ and high values of LRT, WD, LDMC and RL/LA. Negative PC1 values denoted species with low values of LRT and high (less negative) values of Ψ_min_, BWC, SWC and SLA (Fig 1). Because previous studies consider, Ψ_min_ as a proxy of drought tolerance, while rapid leaf loss as a proxy of drought avoidance [7, among others] hereafter we named PC1 as an axis describing a trade-off between tolerance and avoidance and proceeded to use the specie´s scores along PC1 as descriptors of their strategy. PC2 explained only 16% of trait variation, with most covariation among SRL, RB/LB and FRL/LA, though the last two traits also covaried along PC1 (S1 Table). Positive or negative values indicated high or low capability for soil resource acquisition and supply. Thus, hereafter we named PC2 axis as a below-ground continuum of resource acquisition strategies (Fig 1). The functional significance of each multivariate axis is being discussed in the Discussion section.

#### Environmental conditions simulated across pulse scenarios

**Fig 2:**
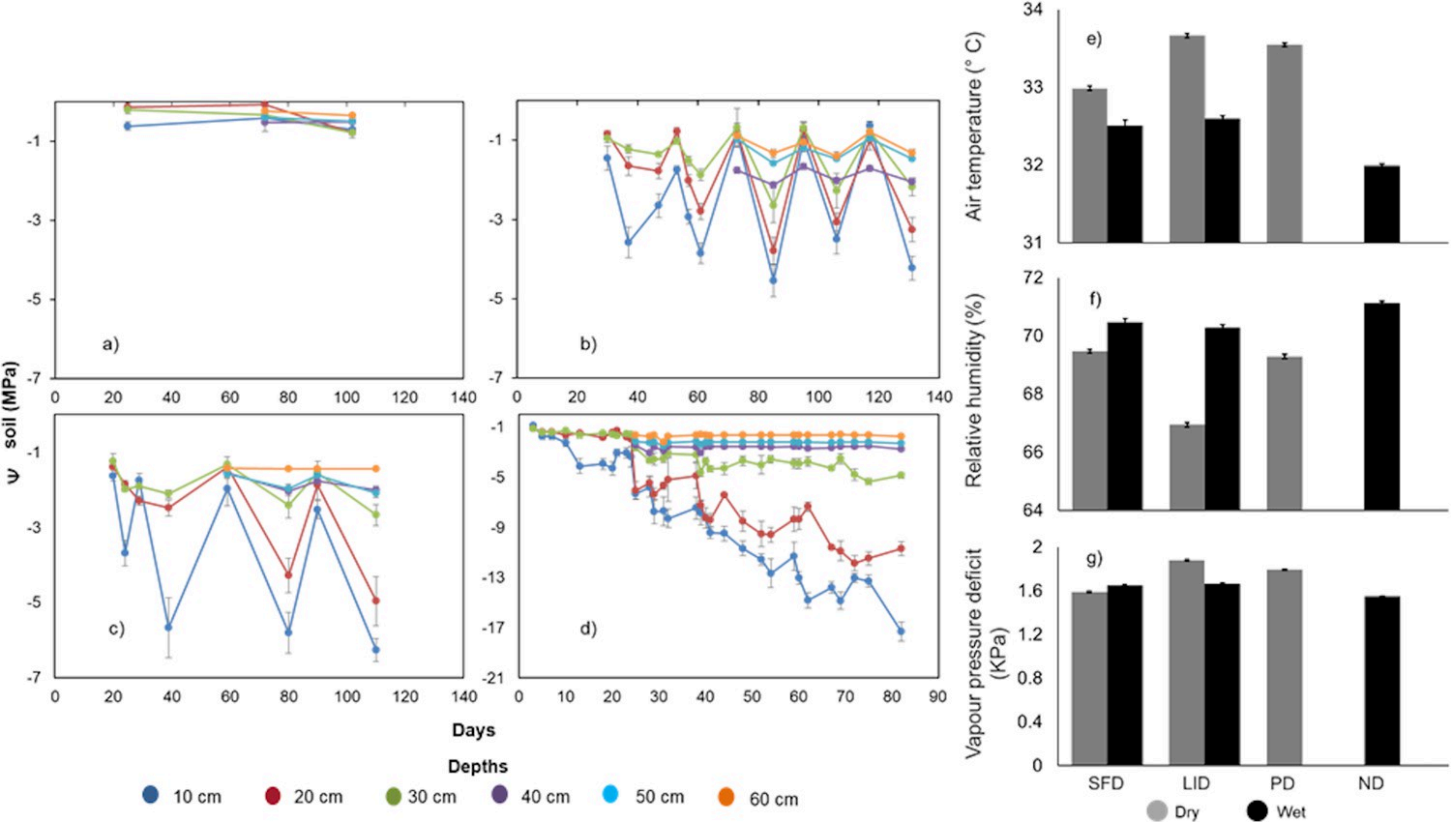
Physical variables during the dry-wet pulse experiments done in the common garden and the greenhouse. I. simulation of dry-wet pulse scenarios in a common garden. Temporal course of soil water potential (mean and se) at different depths for: a) no drought (ND), b) short and frequent drought pulses (SFD; 15 days drought, 10 days wet), c) long and infrequent drought pulses (LID; 25 days drought, 10 days wet) and d) prolonged drought (PD; no wet period). Atmospheric conditions (mean and se) above the plants during the dry and wet periods imposed by the four treatments: (ND, SFD, LID, PD); e) air temperature, f) relative humidity, h) vapour pressure deficit. II. simulation of a dry-wet pulse scenario (SFD) in the greenhouse. i) no drought (ND), j) short frequent drought (SFD; 20 days drought, 10 days wet). Mean and se values at 20 cm depth. Atmospheric conditions shown in j), k), l). All variables measured at the end of the dry and the wet period, see details in methods.

In our filed experiment, atmospheric conditions above the plants were drier and warmer during dry pulses than during wet pulses (Fig 2). The PD treatment had the driest and ND the wettest conditions overall. Soil water potential varied strongly in response to watering, with clear dry-wet pulses that were stronger the longer the pulse and closer to the soil surface (Fig 2). In the ND treatment, soil water potential remained high throughout the experiment, regardless of depth (-0.8 MPa to -0.2 MPa). Near the surface, the SFD soil water potential varied from -0.5 MPa to -4.8 MPa during the wet and dry pulses; in LID, it ranged from -1.3 MPa to -6.0 MPa. In the PD, near the surface the water potential decreased over time, from -0.8 to <-10.0 MPa, but remained stable at -0.9 MPa at depths below 40 cm (Fig 2). In the greenhouse, the short dry-wet pulse simulation generated similar variation in the soil water potential (-0.7 MPa to– 3.8 MPa) (S1 Fig) to that recorded in the field experiment (both evaluated at 20 cm depth), but the atmosphere was drier in the greenhouse (1.59 KPa to 1.61 KPa, and 0.88 KPa to 0.91 KPa, in the greenhouse and field, respectively) (S1 Fig).

### Effects of dry-wet pulse scenarios on seedlings survival and growth in the field

**Fig 3:**
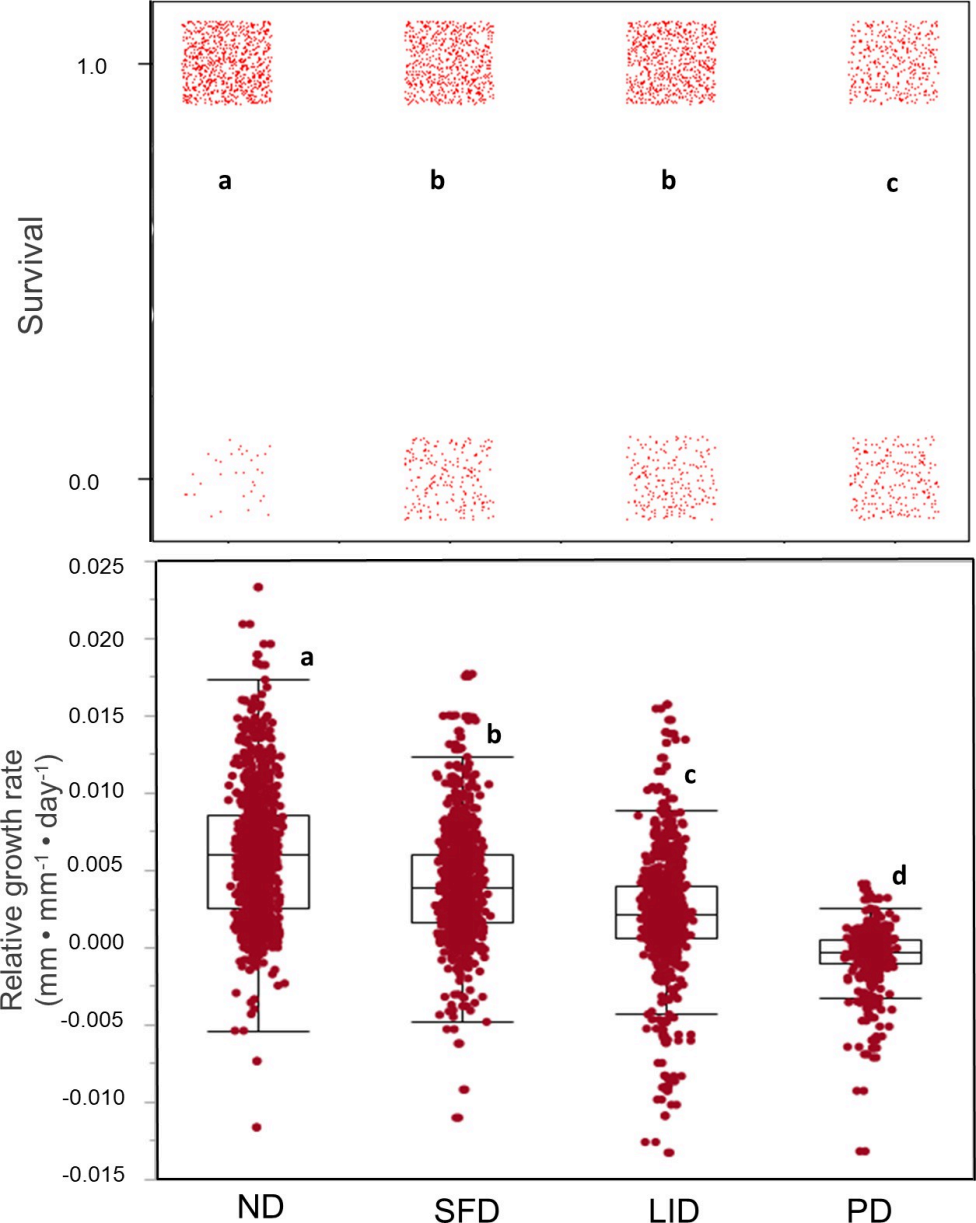
Performance of seedlings of 19 tropical dry forest tree species in response to different dry-wet pulse scenarios in field conditions: a) survival, and b) mean relative growth rate in each dry-wet pulse scenario. Points indicate individual plants, while boxes indicate 25% and 75% percentiles. ND: no-drought; SFD: short and frequent drought pulses, LID long and infrequent drought pulses; and PD: prolonged drought. Different letters indicate significant differences in the mean between dry-wet pulse scenarios at P < 0.05, according to a mixed model including species as a random factor.

Seedling´s responses to dry-wet pulses were evident. During the dry periods they exhibited variable signs of dehydration and leaf loss, while during the wet periods they recovered hydration and produced new leaves. Plant survival and mean relative growth rate varied significantly among the dry-wet pulse scenarios (*X*^2^ = 282.4, 3 df, p < 0.001, *X*^2^ = 282.4, 3 df, p < 0.001 283.5, 3 df, p < 0.001, for survival and RGR, respectively), (Fig 3). Both parameters tended to decrease from wetter (ND) to drier (PD) scenarios; this trend was stronger in RGR than in survival (Fig 3). Both RGR and survival were significantly lower under prolonged droughts, with RGR even showing negative values, in comparison to the ND scenario where all species exhibited their maximum performance.

### Interaction between the dry-wet pulse scenario and functional strategy

#### Field common garden

**Fig 4:**
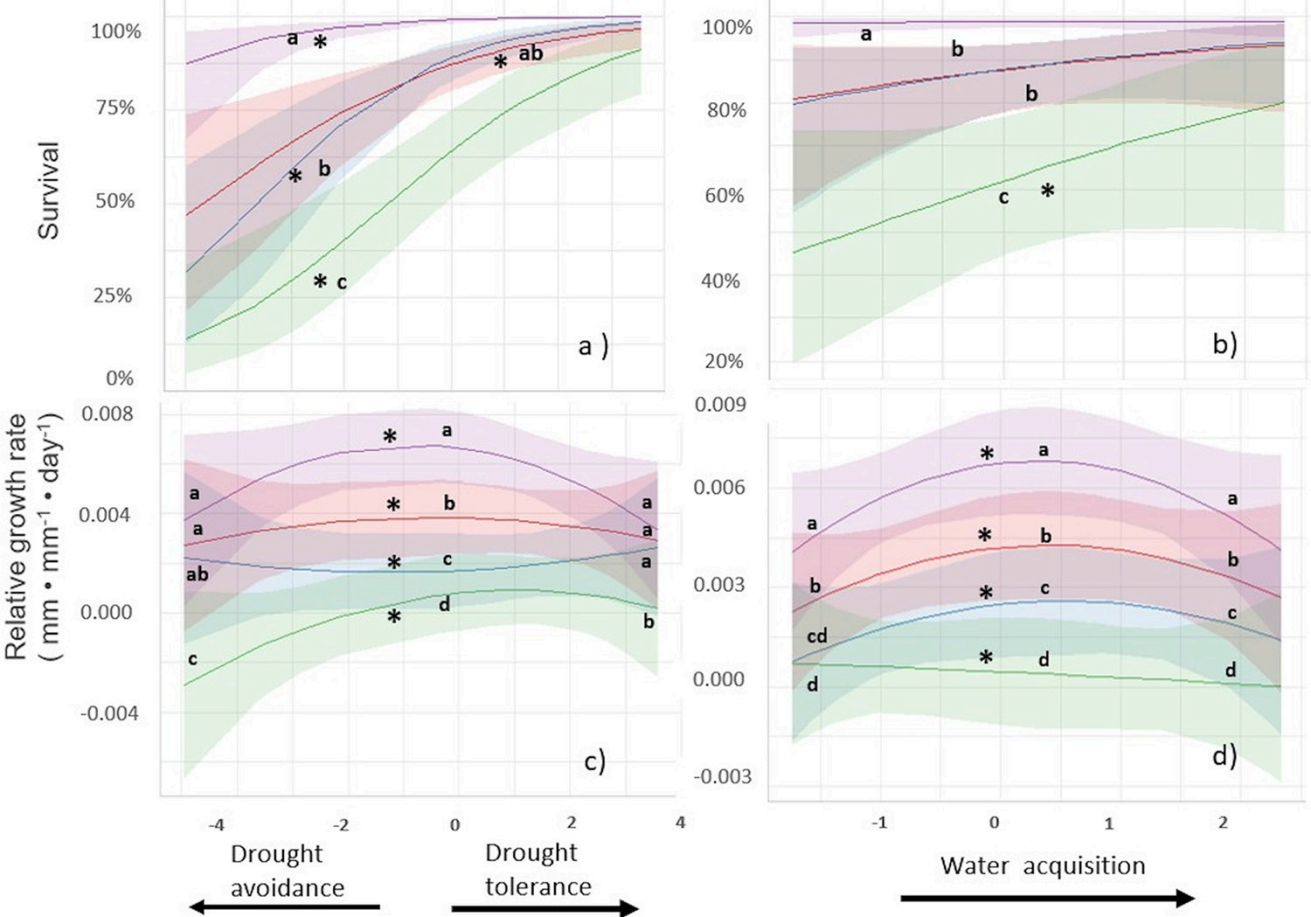
Seedling´s survival and RGR in relation to species’ functional strategies across dry-wet pulse scenarios simulated in field conditions, according to mixed models shown in Tables 2, 3. PC1 scores represent a drought tolerance-drought avoidance continuum, and PC2 scores represent a belowground resource acquisition continuum. Colored shading indicates 95% prediction confidence intervals. * Indicates significant regressions. For a), b), different letters indicate significant differences of slopes between drought pulse scenarios. For c) and d), different letters indicate significant differences between the trends evaluated at three different values of the regressor. For PC1: low (-5), intermediate (0) and high (3.5); for PC2: low (-1.5), intermediate (0.5) and high (2.0).

For the field experiment, the mixed model analyses controlling for initial plant size showed that plant survival was affected by the species’ functional strategy, as described by their PC1 and PC2 scores, in interaction with the dry-wet pulse scenario (Table 3). Under ND conditions, survival did not relate to the PC1 axis, but under drought scenarios, survival monotonically increased with PC1 scores, and this relationship was steeper with increasing length of the drought pulse (Fig 4A and S2 Table). Thus, species with drought tolerant traits (i.e., higher WD, LDMC, LRT values and lower Ψ_min_) exhibited higher survival than species with drought avoidance traits, especially under treatments with longer drought pulses (Fig 4A). Similarly, the species’ ability to capture soil resources, as represented by PC2 scores, did not relate to survival under ND conditions but there was a significant positive relationship under the PD scenario (Fig 4B, Table 3 and S2 Table).

**Table 3:** Effects of species’ functional strategy (PC1 or PC2 scores) and dry-wet pulse scenario on survival, while controlling by plant height, in seedlings of 19 TDF species grown in a field common garden experiment. (A) generalized linear mixed model for survival against PC1 (R^2^_m_ = 0.27; R^2^_c_ = 0.36) and B) generalized linear mixed model for survival against PC2 (R^2^_m_ = 0.19; R^2^_c_ = 0.39). *X*^2^ values correspond to Wald Type III test statistics. Each model considered 3463 individuals.

Continuum of functional strategies	Predictors	*X* ^*2*^	DF	Pr(>Chisq)
A) PC1 (avoidance-tolerance)	Height	10.84	1	**<0.001**
	PC1	23.88	1	**<0.001**
	Dry-wet pulse scenario	326.33	3	**<0.001**
	PC1*Dry-wet pulse scenario	10.34	3	**0.016**
B) PC2 (water acquisition)	Height	14.21	1	**<0.001**
	PC2	1.35	1	0.24
	Dry-wet pulse scenario	322.28	3	**<0.001**
	PC2*Dry-wet pulse scenario	21.12	3	**<0.001**

RGR was affected by the species’ functional strategy, both as described by PC1 and PC2 axes, and these effects varied with the drought pulse scenario (Table 4 and S3 Table). Under ND conditions, RGR showed a concave relationship where plants with intermediate PC1 values grew the most (Fig 4C and S4 Table). In contrast, this relationship was nearly null under SFD and convex under the PD scenario (Fig 4C and S4 Table), indicating a strong RGR decrease among the species with intermediate PC1 scores relative to the species with extreme PC1 values (Fig 4C). Finally, under the prolonged drought, RGR was highest for species with high PC1 scores, the tolerant species and lowest for the avoider species with low PC1 values, resulting in a positive linear relationship (Fig 4C and S4 Table). RGR also varied with PC2, showing a concave trend under ND, SFD, where species with intermediate abilities to capture soil resources (PC2 values) exhibited the highest growth rates (Fig 4D and S5 Table). In the PD treatment, however, there was a negative linear relationship in which the species with low ability to capture soil resources exhibited the highest RGR (Fig 4D and S5 Table). Although plants hardly grew or even decreased in size under the most drastic drought scenario, the least affected species showed a tolerant strategy or a soil resource conservative strategy (Fig 4C and 4D).

**Table 4:** Effects of species functional strategy (PC1 or PC2 scores) and dry-wet pulse scenario on RGR, while controlling by plant height, in seedlings of 19 TDF species growing in a field common garden experiment. A) quadratic mixed model for RGR against PC1 (R^2^_m =_ 0.34_;_ R^2^_c_ = 0.60). B) quadratic mixed model for RGR against PC2 (R^2^_m_ = 0.35; R^2^_c_ = 0.61). *X*^2^ values correspond to Wald Type III test statistics. Each model considered 2862 individuals.

Continuum of functional strategies	Predictors	*X* ^*2*^	DF	Pr(>Chisq)
A) PC1 (avoidance-tolerance)	Height	223.01	1	**<0.001**
	Dry-wet pulse scenario	1160.61	4	**<0.001**
	Polynomial (PC1^2^)	0.78	2	0.68
	Dry-wet pulse scenario*poly (PC1^2^)	93.90	6	**<0.001**
B) PC2 (water acquisition)	Height	238.08	1	**<0.001**
	Dry-wet pulse scenario	1051.35	3	**<0.001**
	Polynomial (PC2^2^)	1.26	2	0.53
	Dry-wet pulse scenario*poly (PC2^2^)	37.19	6	**<0.001**

#### Greenhouse

**Fig 5:**
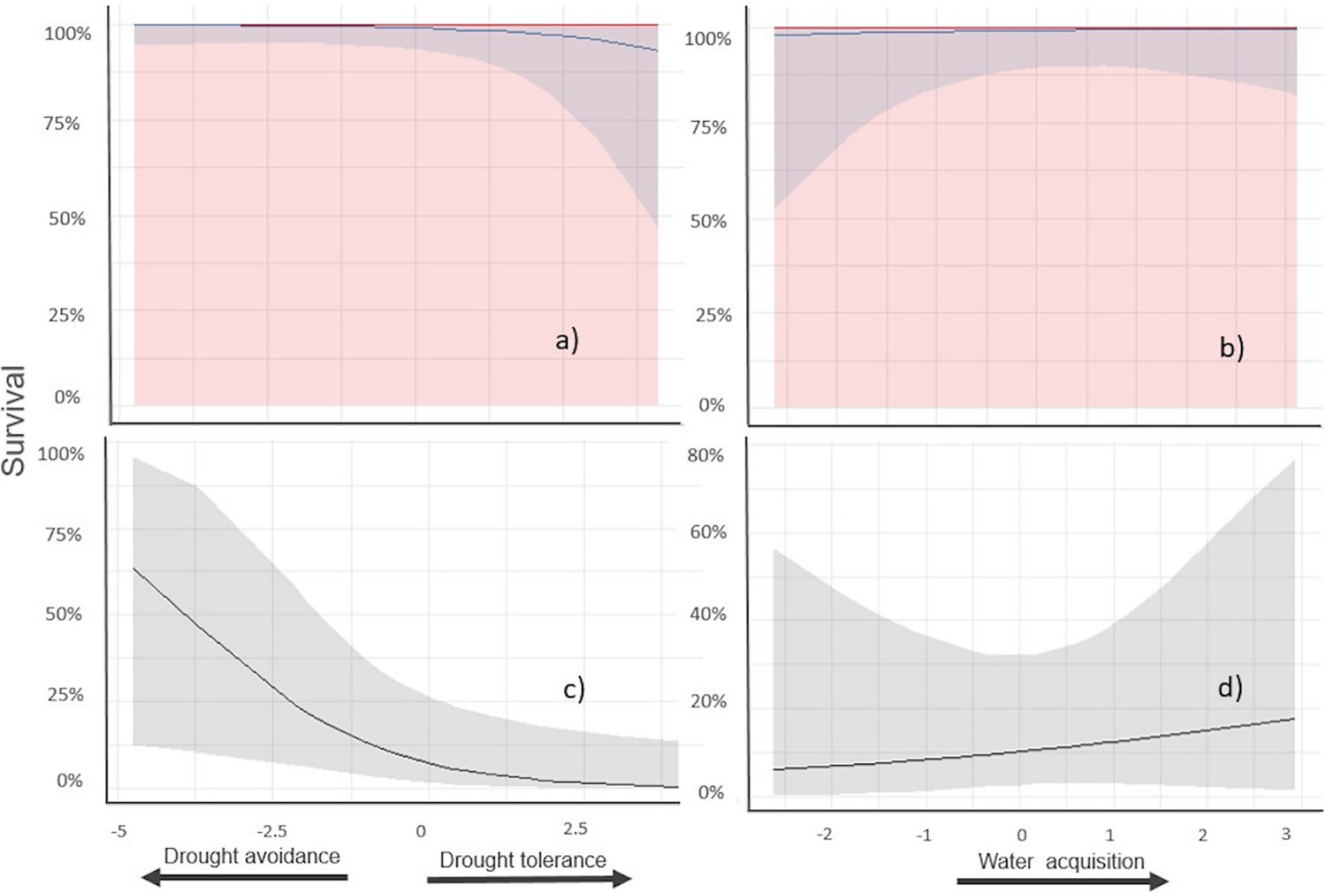
Seedling´s survival in relation to plant functional strategies (PC1, PC2 species scores) under greenhouse conditions. Panels a), b) plants subject to a simulated dry-wet pulse and non-drought scenarios, respectively. Trend lines and confidence intervals from mixed models are shown (red no-drought, blue dry-wet pulse). a) survival vs. PC1 (*X*^*2*^ < 0.01, p > 0.99 for dry-wet pulse or no-drought scenarios), b) survival vs. PC2 (*X*^*2*^ < 0.01, p > 0.99 for dry-wet pulse or no-drought scenarios). Panels c), d) survival after drought-provoked dieback. Trend lines and confidence intervals from mixed models are shown. c) trends against PC1 species scores describing a drought avoidance vs. tolerance continuum (*X*^*2*^ = 4.61 *p <* 0.05), d) trends against PC2 species scores describing an acquisitive resource use continuum (*X*^*2*^ = 0.20 *p =* 0.64).

In the greenhouse, mortality was practically null in both the control (no drought) and the short dry-pulse scenario regardless of plant strategy (*X*^2^ < 0.01, P > 0.99 for any PC1 or PC2 effect) (Fig 5A and 5B and S6 Table). On the other hand, in the prolonged drought greenhouse experiment, survival, after drought-provoked dieback, was negatively related to PC1 (*X*^2^ = 4.61, P < 0.05), while was invariant to PC2 (*X*^2^ = 0.21, P > 0.64) (Fig 5C and 5D and S7 Table).

## Discussion

### The continuum of plant functional strategies among TDF seedlings

The strong covariation detected among most of the leaf, stem, and whole plant traits along one multivariate axis explaining 44% of trait variation in our data set, concurs with the idea that a trade-off between drought avoidance and drought tolerance is a major axis organizing functional strategies in TDFs [12, 13, 24]. Among seedlings of TDF´s, the joint ability to rapidly reduce foliar area (and water loss) and store water suggests a doble mechanism to avoid or delay the effects of drought [12]. The high values of critical mid-day water potential (Ψ_min_) associated to avoiders in our study and in others [28], suggests that such avoiding mechanisms are indeed effective to maintain water status during droughts (but see [52]), albeit these species tend to have a xylem vulnerable to cavitation [13]. In our data set, *Ipomoea wolcottiana*, *Ceiba aesculifolia* and *Spondias purpurea* represent extreme avoiders. On the other hand, among tardly-deciduous species the observation of very low critical water potentials at mid-day, suggests a high capacity of tissues to maintain functioning well into the drought period [23]. Among these species, the presence of tissues (leaves, and stems) with high density (high dry mass content), has been associated with reduced lumen xylem vessels, which low vulnerability to cavitation may also relate to reinforced thick cell walls or fiber layers [53]. Species as *Mimosa arenosa*, *Caesalpinia platyloba* and *Apoplanesia panniculata*, are examples of the tolerant end. In principle, those species seated far from the extremes, that do not behave as rapid avoiders or stoic tolerants, are expected to be more vulnerable to drought. (e.j. *Crescentia alata* and *Guazuma ulmifolia*). However, the reduced investment in storage or dense tissues may allow them to allocate more to the capacity of water acquisition and use, fueling higher growth rates when water is available [54].

Interestingly, in our data set, root traits do not fully covary along the avoidance vs. tolerance continuum (Fig 1), suggesting that the ability of plants to capture soil resources may be partly uncoupled from plant strategies to deal with intense drought, as suggested by the high loadings of such traits along PC2. Such uncoupling of root and drought resistance strategies has been observed among young plants of other neotropical forests; Bolivia [55], Colombia [56] and Mexico [57, 58, but see 59], and may reflect the occurrence of multiple factors shaping root trait variation [60]. Whether strategies to deal with drought and to acquire resources are tightly coordinated or independent, is still an active research question [58]. In this study given the strong coordination detected among leaf, stem and root traits along the drought avoidance vs drought tolerance continuum, and the prevalence of it across multiple studies, we decided to focus principally on such a continuum to discuss the interplay with the dry-wet pulse scenarios.

### Seedling´s performance is affected by the dry-wet pulse scenario

As expected, the drought pulse scenarios simulated in our field study negatively affected both sapling growth and survival. The decrease in plant performance over the course of the drought pulse, suggests a cumulative effect of drought on plant condition. The plants did not fully recover from the negative effects of drought during the 10-day wet period simulated here. Because in Chamela repetitive dry spells are common, dry-wet pulses may impose an important ecological filter at early life stages, particularly during dry years when long dry-spells and short wet periods are more common [22]. Repetitive dry spells are also common in other tropical dry forests [10], but frequently studies have only quantified responses to single intense droughts [33, 61]. Repetitive droughts and their negative effects on vegetation are projected to occur at larger ecological and temporal scales, and tropical forests are at particular risk because recovery of productivity between long droughts would not compensate for losses [62, 63]. Because droughts are expected to increase in duration and frequency with climate change [18], future studies assessing the impacts of the complex dry-wet pulse scenarios on recruitment processes should consider both the long-term joint variation of recruitment and water availability, and the experimental responses of plants to pulses, as modeled under diverse drought frequency and duration scenarios. Here we contribute by examining recovery responses to increasing drought duration, a relatively simple but key factor.

### Different dry-wet pulse scenarios favor different strategies to resist drought

We predicted that short frequent droughts would disfavor survival of the drought avoiders (since the rapid loss and production of leaves would lead to cumulative carbon unbalance) and favor tolerant species that are able to retain leaves and keep carbon gain longer during droughts. Additionally, we predicted that prolonged droughts would act against tolerant species, since they eventually reach massive cavitation. Contrary to these expectations, our results indicated that under all drought scenarios tolerant plants have the advantage over avoider plants, and that this advantage increases with the drought pulse duration (Fig 4). This result is intriguing. Although in this study we did not aim to disentangle the mechanisms of drought-induced death [64], the increasing disadvantage of avoiders with drought duration suggest that rapid leaf loss is not enough to prevent cavitation of their vulnerable vessels [52, 28]. This results in a higher cumulative effect of cavitation across dry-wet pulses, especially when there are no periods of hydration recovery. However, the observation of a much higher prevalence of sucking herbivores and necrosed tissue among the drought avoider species (unpublished), may suggest that lower survival in those species results from higher burden of attack on plants with elevated contents of water and non-structural carbohydrates [65–68], valuable resources for insects and pathogens during dry periods [67]. This is also supported by the fact that even in the no-drought treatment, drought avoiders exhibited some survival disadvantage (Fig 4) and had higher prevalence of pathogen attack (unpublished). The role of natural enemies in drought experiments with tropical young plants has also been documented elsewhere [69].

The survival advantage of drought-tolerant over drought-avoider plants mediated by natural enemies also seems to be supported by our complementary experiments under greenhouse conditions. Under controlled conditions, a short dry-wet pulse scenario did not favor any strategy to deal with drought. Secondly, with progressive soil desiccation, the avoider species took much longer to reach massive cavitation of the main stem (255 days and 49 days to reach 30% dieback, for avoiders and tolerators, respectively). Third, when rewatering, the survival trend reversed; avoider species survived better than tolerant species (Fig 5). Although general mechanisms of use of water and carbohydrate storages in trees still wait to be discerned [70, 71], previous studies of TDF seedlings in Chamela [28] demonstrated that readily deciduous species with high water reserves can prevent massive cavitation by maintaining high stem water potential much longer than tardily deciduous species with low reserves, which despite maintaining leaves long into drought periods at some point suffer catastrophic cavitation. Also, the potential role of carbohydrate reserves on maintaining stem water potential during droughts has been reported for seedlings of other tropical forests [15, 72]. Together, our field-common garden and greenhouse experiments suggest that drought avoider species are physiologically better equipped to survive long droughts than drought tolerant species. However, under field conditions, other factors acting against drought avoiders such as natural enemies may reverse the ranking of drought response among species. The survival advantage of tolerant species over avoiders after intense droughts coincides with previous studies evaluating adult trees in tropical forests [73, 74]. Our study points to the hypothesis that such an advantage is not solely related to a xylem with low vulnerability to cavitation, but to the interplay with natural enemies, a hypothesis whose generality awaits testing.

We predicted that under no-drought conditions, species with intermediate functional traits will grow faster because they invest less in productive tissues (e.g., storage, dense tissues). However, because fast-growing plants are neither extremely avoidant nor tolerant, they are expected to suffer more severe cumulative loss of conducting capacity or carbon balance [28] and lose their advantage in scenarios with long dry periods. Our results coincide with these predictions. In fact, the observed preference of fast-growing species for relatively humid habitats in the dry forest in Chamela [57], suggests a relatively wet spatial-temporal niche where they can coexist. The rapid drop in water availability and increase in VPD we imposed with the progressive drought scenario strongly restricted growth for all surviving plants, particularly for drought avoiders, which exhibited mostly size decreases. This result is consistent with the observation that in the tropical dry forests, drought-tolerant trees can sustain slow growth long during the dry season [75], although in other reports their growth advantage during intense droughts is less clear [74]. The causes of large decreases in the height of avoider plants are unclear, though apical death of the main stem may reflect an interplay between massive cavitation and pathogen attacks, as discussed before for survival.

In our study, the examination of the relationships between root traits and drought performance suggested that different dry-wet pulse scenarios may shape different resource use strategies belowground. For example, a large investment in surface capture belowground favored survival under prolonged drought while was irrelevant under no-drought. Albeit many studies have suggested the importance of a large root surface capture to maintain plants’ hydration under intense drought [65, 75], our experimental test is one of the few done with tropical tree seedlings in natural settings. Why such survival advantage did not express under greenhouse conditions is intriguing. The reduced soil volume in pots may limit the benefits of increasing root absorption surface, making survival more dependent on the mechanisms to tolerate or delay drought effects, as discussed before.

Strikingly, those species that invested more in surface capture belowground did not grow faster in any field scenario. Indeed, species with intermediate strategies grew faster under no-drought, while high investors in root surface were at disadvantage under intense drought. In this extreme scenario, while in general plants hardly grew or decreased, those species with elevated root surface investments suffered the most. The observation of higher rates of herbivory and pathogen attack among species with high root surface investments (personal obs.), suggests that these patterns of growth response may be driven by a potential trade-off between defense and acquisition capacity [76]. Overall, our results suggest that species with a disproportionate investment in root acquisitive capacity may succeed in long dry pulses because they have higher water provision and hydration maintenance, but at the direct or indirect cost of carbon losses and growth. A better understanding of the role of root traits and strategies to deal with different dry-wet pulse scenarios might need the consideration of other traits as root depth and the association with mycorrhizae.

## Conclusions

The fate of plants in many terrestrial habitats with temporally heterogeneous water availability depends on both drought resistance and recovery, thus potentially shaping plant communities. We showed evidence that plant functional traits of young plants affect species’ performance under simulated drought-water pulse scenarios, and that different drought-water pulses may favor different plant functional strategies among TDF tree species. Albeit evidence of certain level of spatial hydric differentiation among species has been reported in TDF [77], our results point that the temporal heterogeneity in water regime may also contribute to the local coexistence of radically different tree strategies in the same forest. In addition, the finding of differential sensitivity of tree species to frequency and length of repeated droughts in our study, calls for the need to incorporate the interdependent responses of trees to repeated dry-wet pulses, in the efforts to anticipate how plants communities will be shaped by the increasing temporal variability in water regimes with climate change. It is important to note that our simulations were simple, maintaining a constant recovery phase duration while varying the drought period. A better understanding of the response to drought-water pulses and identifying potential thresholds will require testing using variation in both pulse phases and explicit analysis of plasticity in plants’ responses.

## Supporting information

S1 Text Measurement of plant functional traits. (DOCX)

S2 Text Measurement of physical variables in the field and greenhouse experiments. (DOCX)

S1 Fig Physical variables in the greenhouse dry-wet pulse experiment. (TIF)

S1 Table PCA loadings of functional traits for saplings of 18 tree tropical dry forest species. (DOCX)

S2 Table Parameters derived from mixed GLM models for survival in a field experiment. (DOCX)

S3 Table Parameters derived from mixed GLM models for relative growth rate (RGR) in a field experiment. (DOCX)

S4 Table Comparisons of predicted RGR values across three contrasting values of PC1, among dry-wet pulse scenarios in the field experiment. (DOCX)

S5 Table Comparisons of predicted RGR across three contrasting values of PC2, among dry-wet pulse scenarios in the field experiment. (DOCX)

S6 Table Effects of species functional strategy (PC1 or PC2 scores) and dry-wet pulse scenario on survival in greenhouse conditions. (DOCX)

S7 Table Effects of species functional strategy (PC1 or PC2 scores) on survival after individuals suffered 30% dieback under greenhouse conditions. (DOCX)
