# Correction: Different dry-wet pulses favor different functional strategies: A test using tropical dry forest tree species

**DOI:** 10.1371/journal.pone.0323025

**Published:** 2025-04-22

**Authors:** Flor Vega-Ramos, Lucas Cifuentes, Fernando Pineda-García, Todd Dawson, Horacio Paz

The following information is missing from the Funding statement: H. Paz was also supported by a PASPA, UNAM sabbatical fellowship.
