# Supplementary material for: Different dry-wet pulses favor different functional strategies: A test using tropical dry forest tree species
Source: PLoS One. 2024 Dec 3;19(12):e0309510. doi: 10.1371/journal.pone.0309510 (PMC11614228; doi:10.1371/journal.pone.0309510)
Supplement: S1 Text — (DOCX) [file pone.0309510.s001.docx]

**S1 Text. Measurement of plant functional traits**

Three-month-old plants were cropped and excised into leaves, stems and roots, to obtain leaf area (LT) and the minimum photosynthetic unit size (MFS) from digital images. One 3-cm section was obtained from the stems, rehydrated for 12 hours, and excised to separate the wood from the bark. The root system was carefully washed and divided into fine roots (i.e., absorption roots, ≤ 2 mm in diameter), and thick roots (i.e., storage roots, >2mm diameter) (Pineda-García et al., 2011). The total fine root length (FRL) was obtained from digital images of fresh tissues analyzed with WinRhizo software (Regent Instruments, Inc, Neplean, ON, Canada). Finally, all tissues were oven-dried for 72 h at 70°C and weighed to obtain the total leaf biomass (LB), specific leaf area (SLA), leaf dry matter content (LDMC), leaf water content (LWC), wood density (WD), stem water content (SWC), bark water content (BWC), total root biomass (RB), fine root biomass (FRB), specific root length (SRL) and root water content (RWC). Additionally, we calculated the FRB:LB, FRL:LA and RB:LB ratios. The leaf retention time, here defined as the time necessary to lose 50% of standing leaf area, was determined by monitoring the percentage of initial leaf area throughout the prolonged drought treatment for 15 individuals per species, then fitting a decaying non-linear model for each species following (Méndez-Alonzo et al., 2012). The minimum mid-day leaf water potential was measured during the experiment and during the prolonged drought treatment just before saplings dropped their leaves, measuring one non-damaged leaf with a pressure chamber, model 1505D (PMS Instruments company, Albany OR; USA).
