# Supplementary material for: Different dry-wet pulses favor different functional strategies: A test using tropical dry forest tree species
Source: PLoS One. 2024 Dec 3;19(12):e0309510. doi: 10.1371/journal.pone.0309510 (PMC11614228; doi:10.1371/journal.pone.0309510)
Supplement: S2 Text — (DOCX) [file pone.0309510.s002.docx]

**S2 Text. Measurement of physical variables in the field and greenhouse experiments**

Field common garden

To characterize the dry-wet pulses generated by the watering treatments, we monitored the temperature and humidity of air and water potential of the soil at different depths throughout the experiment. Temperature and relative humidity were recorded for each treatment every 30 minutes during the experimental period using eight HOBO Pro V2 units (two sensors in two sub-plots per treatment), located 50 cm above the soil surface. The vapor pressure deficit (VPD, KPa) was calculated by using the Penman-Monteith equation:$VPD =[PVsat-PVair]$ (McNaughton & Jarvis, 1984). In each treatment, the water content of the 10cm – 60cm soil column was monitored by taking soil cores from each sub-plot. In the no-drought treatment (control), soil cores were taken at the beginning, middle, and end of the experiment. In the short and long drought treatments, soil cores were taken at the end of the wet periods and at the end of the dry periods. Several soil cores were taken in the prolonged drought scenario when trees began to clearly exhibit changes in their wilting condition. Gravimetric water content was converted to soil water potential (Mpa) by using a water-release curve calculated by desiccating soil samples in lab conditions and using a WP4-C Dewpoint Potentiometer (Meter, Inc., USA).

Greenhouse

Atmospheric conditions and soil water potential were monitored throughout the experiment. Soil water content was measured at the end of the dry and the wet pulses in 10 pots of each species (180 pots) at 20 cm depth, Soil water content was measured at the end of the dry and the wet pulses in 10 pots of each species (180 pots) at 20 cm depth by laterally inserting a TDR probe (Wet, Delta T, EUA). Atmospheric conditions above the seedlings in one block were logged every 30 min during the whole experiment using HOBO U23 proV2 loggers (ONSET, EUA). Mean daily air temperature and relative humidity were obtained, and VPD was calculated by applying the Penman-Monteith equation (McNaughton & Jarvis, 1984). At the end of the experiment, we evaluated the survival of each species.
