# Supplementary figures and images for: Different dry-wet pulses favor different functional strategies: A test using tropical dry forest tree species
Source: PLoS One. 2024 Dec 3;19(12):e0309510. doi: 10.1371/journal.pone.0309510 (PMC11614228; doi:10.1371/journal.pone.0309510)

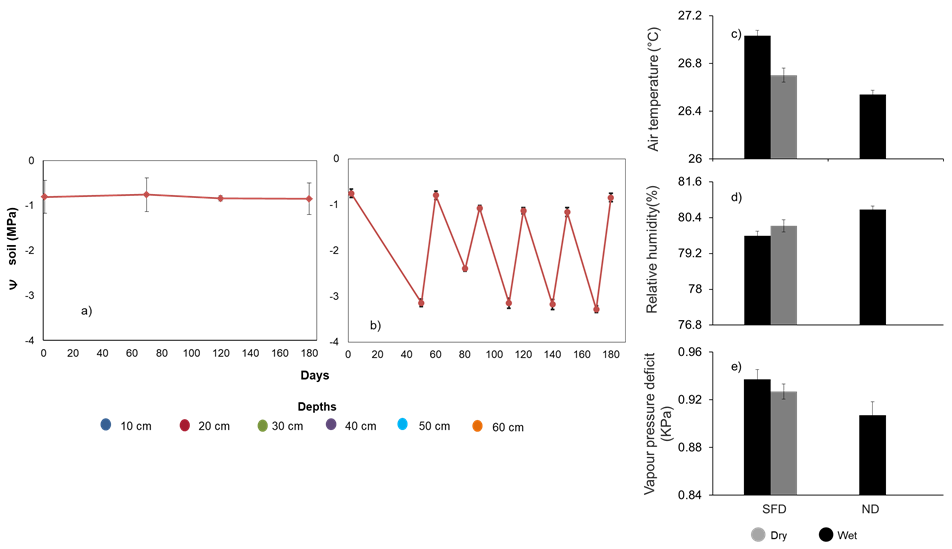

Supplement: S1 Fig — (TIF) [file pone.0309510.s003.tif]
