# Supplementary material for: Different dry-wet pulses favor different functional strategies: A test using tropical dry forest tree species
Source: PLoS One. 2024 Dec 3;19(12):e0309510. doi: 10.1371/journal.pone.0309510 (PMC11614228; doi:10.1371/journal.pone.0309510)
Supplement: S1 Table — (DOCX) [file pone.0309510.s004.docx]

S3 Table. PCA loadings of functional traits for saplings of 18 tree tropical dry forest species. BWC: Bark water content, FRB/LB: Fine root biomass/total leaf biomass, FRL/LA: Fine root length/ total leaf area, LDMC: Leaf dry matter content, LRT: Leaf retention time, MPS: Minimum photosynthetic unit size, RB/LB: Total root biomass/total leaf biomass, RWC: Root water content, SLA: Specific leaf area, SRL: Specific root length, SWC: Stem water content, VRER: Vertical root elongation rate, WD: Wood density, Ψmin: Minimum leaf water potential.

|  | **Attribute** | **PC1** | **PC2** |
| --- | --- | --- | --- |
| **Eigenvalues** |  | 5.8018 | 2.0936 |
| **%** |  | 44.629 | 16.104 |
| **Eigenvectors** | BWC | -0.36248 | 0.11723 |
|  | FRB/LB | 0.20927 | 0.46243 |
|  | FRL/LA | 0.28212 | 0.45196 |
|  | LDMC | 0.35259 | -0.10919 |
|  | LRT | 0.31592 | -0.08991 |
|  | MPS | -0.15732 | 0.15733 |
|  | RB/LB | -0.13362 | 0.43283 |
|  | SLA | -0.23932 | 0.19872 |
|  | SRL | 0.1946 | 0.48593 |
|  | SWC | -0.35056 | -0.00687 |
|  | VRER | 0.06937 | -0.20533 |
|  | WD | 0.36668 | -0.08268 |
|  | Ψ_min_ | 0.34812 | -0.1067 |
