# Supplementary material for: Different dry-wet pulses favor different functional strategies: A test using tropical dry forest tree species
Source: PLoS One. 2024 Dec 3;19(12):e0309510. doi: 10.1371/journal.pone.0309510 (PMC11614228; doi:10.1371/journal.pone.0309510)
Supplement: S2 Table — (DOCX) [file pone.0309510.s005.docx]

S5 Table.- Table of parameters derived from mixed GLM models for survival in a field common garden as a function of a) PC1 species scores and dry-wet pulse scenario, and b) PC2 species scores and the dry-wet pulse scenario.

|  | Predictors | Estimate | | Z value | Pr(>\|z\|) |
| --- | --- | --- | --- | --- | --- |
| 1. PC1 | (Intercept) | | 1.5481 | 10.963 | **<0.0001** |
|  | Initial height | | 0.006886 | 3.293 | **<0.0010** |
|  | PC1 | | 0.149048 | 2.586 | **0.0097** |
|  | SFP | | -1.036281 | -10.967 | **<0.0001** |
|  | LIP | | -1.022575 | -10.793 | **<0.0001** |
|  | PD | | -1.794413 | -15.912 | **<0.0001** |
|  | PC1*SFP | | 0.058993 | 1.507 | 0.1318 |
|  | PC1*LIP | | 0.109062 | 2.756 | **0.0058** |
|  | PC1*PD | | 0.128213 | 2.743 | **0.0061** |
| 1. PC2 | (Intercept) | | 1.64642 | 8.833 | **<0.0001** |
|  | Initial height | | 0.008231 | 3.77 | **0.0002** |
|  | PC2 | | -0.067099 | -0.483 | 0.6292 |
|  | SFP | | -1.155319 | -13.162 | **<0.0001** |
|  | LIP | | -1.161051 | -13.269 | **<0.0001** |
|  | PD | | -1.944265 | -18.004 | **<0.0001** |
|  | PC2*SFP | | 0.233929 | 3.743 | **0.0002** |
|  | PC2*LIP | | 0.243051 | 3.883 | **0.0002** |
|  | PC2*PD | | 0.302113 | 4.198 | **<0.0001** |
