# Supplementary material for: Different dry-wet pulses favor different functional strategies: A test using tropical dry forest tree species
Source: PLoS One. 2024 Dec 3;19(12):e0309510. doi: 10.1371/journal.pone.0309510 (PMC11614228; doi:10.1371/journal.pone.0309510)
Supplement: S3 Table — (DOCX) [file pone.0309510.s006.docx]

S6 Table.- Table of parameters derived from GLM mixed models for relative growth rate (RGR) in a field common garden as a function of a) PC1 species scores and dry-wet pulse scenario, and b) PC2 species scores and the dry-wet scenario pulse scenario. The model includes linear and quadratic components of the regressor (PC1 and PC2).

|  | Predictors | Estimate | t value | Pr(>\|t\|) |
| --- | --- | --- | --- | --- |
| 1. PC1 | Intercept | 6.90E-03 | 12.71 | **<0.0001** |
|  | Initial height | -7.00E-05 | -14.93 | **<0.0001** |
|  | SFP | -2.13E-03 | -15.18 | **<0.0001** |
|  | LIP | -3.71E-03 | -27.05 | **<0.0001** |
|  | PD | -5.44E-03 | -25.30 | **<0.0001** |
|  | PC1 | -5.37E-03 | -0.17 | 0.8701 |
|  | (PC1)^2^ | -5.91E-02 | -1.93 | 0.0731 |
|  | SFP*PC1 | 9.09E-03 | 1.05 | 0.3000 |
|  | LIP*PC1 | 1.20E-02 | 1.40 | 0.1611 |
|  | PD*PC1 | 5.65E-02 | 3.59 | **<0.0001** |
|  | SFP*(PC1)^2^ | 4.06E-02 | 4.58 | **<0.0001** |
|  | LIP*(PC1)^2^ | 7.34E-02 | 8.54 | **<0.0001** |
|  | PD*(PC1)^2^ | 2.33E-02 | 1.58 | 0.1221 |
| 1. PC2 | Intercept | 6.87E-03 | 12.73 | **<0.0001** |
|  | Initial height | -7.14E-05 | -15.43 | **<0.0001** |
|  | SFP | -2.16E-03 | -15.26 | **<0.0001** |
|  | LIP | -3.74E-03 | -27.16 | **<0.0001** |
|  | PD | -5.15E-03 | -25.61 | **<0.0001** |
|  | PC2 | 1.46E-02 | 0.45 | 0.6585 |
|  | (PC2)^2^ | -5.30E-02 | -1.79 | 0.0922 |
|  | SFP*PC2 | 2.23E-03 | 0.27 | 0.7881 |
|  | LIP*PC2 | 5.75E-03 | 0.72 | 0.4729 |
|  | PD*PC2 | -2.76E-02 | -2.68 | **0.0073** |
|  | SFP*(PC2)^2^ | 1.84E-02 | 2.24 | **0.0251** |
|  | LIP*(PC2)^2^ | 2.40E-02 | 3.01 | **0.0028** |
|  | PD*(PC2)^2^ | 5.20E-02 | 5.12 | **<0.0001** |
