# Supplementary material for: Different dry-wet pulses favor different functional strategies: A test using tropical dry forest tree species
Source: PLoS One. 2024 Dec 3;19(12):e0309510. doi: 10.1371/journal.pone.0309510 (PMC11614228; doi:10.1371/journal.pone.0309510)
Supplement: S4 Table — (DOCX) [file pone.0309510.s007.docx]

S7 Table.- Contrasts between predicted RGR values calculated by the GLMM as a function of PC1, for the four dry-wet pulse scenarios simulated in the field. We compared RGR at three values of PC1 that describe the three strategies of the functional continuum: -5 for avoider species, 0 for exploiter species and 3.5 for tolerant species.

|  | PC1= -5.0 | | | PC1= 0.0 | | | PC1= 3.5 | | |
| --- | --- | --- | --- | --- | --- | --- | --- | --- | --- |
|  | Estimate | T | P | Estimate | T | P | Estimate | T | P |
| ND-SFP | 0.0010 | 1.84 | 0.2569 | 0.0028 | 14.01 | **<0.0001** | 0.0005 | 1.30 | 0.5654 |
| ND-LIP | 0.0014 | 2.79 | **0.0269** | 0.0049 | 25.41 | **<0.0001** | 0.0008 | 2.29 | 0.1006 |
| ND - PD | 0.0066 | 6.13 | **<0.0001** | 0.0058 | 19.45 | **<0.0001** | 0.0032 | 6.95 | **<0.0001** |
| SFP- LIP | 0.0005 | 0.82 | 0.8454 | 0.0021 | 10.15 | **<0.0001** | 0.0004 | 0.94 | 0.7839 |
| SFP-PD | 0.0057 | 5.14 | **<0.0001** | 0.0030 | 9.76 | **<0.0001** | 0.0027 | 5.80 | **<0.0001** |
| LIP-PD | 0.0052 | 4.75 | **<0.0001** | 0.0009 | 2.96 | **0.0164** | 0.0024 | 5.11 | **<0.0001** |
