# Supplementary material for: Different dry-wet pulses favor different functional strategies: A test using tropical dry forest tree species
Source: PLoS One. 2024 Dec 3;19(12):e0309510. doi: 10.1371/journal.pone.0309510 (PMC11614228; doi:10.1371/journal.pone.0309510)
Supplement: S5 Table — (DOCX) [file pone.0309510.s008.docx]

S8 Table.- Contrasts between predicted RGR values calculated by the GLMM as a function of PC2, for the four dry-wet pulse scenarios simulated in the field. We compared RGR values at three values of PC2 that describe the three strategies of the functional continuum: -1.5 for water-adquisitive species, 0.5 for intermediate species, and 2 for water conservative species.

|  | PC2=-1.5 | | | PC2= 0.5 | | | PC2= 2 | | |
| --- | --- | --- | --- | --- | --- | --- | --- | --- | --- |
|  | Estimate | T | P | Estimate | T | P | Estimate | T | P |
| ND-SFP | 0.0019 | 8.15 | **<0.0001** | 0.0025 | 11.13 | **<0.0001** | 0.0017 | 5.99 | **<0.0001** |
| ND-LIP | 0.0035 | 15.23 | **<0.0001** | 0.0042 | 18.84 | **<0.0001** | 0.0031 | 11.13 | **<0.0001** |
| ND - PD | 0.0039 | 12.49 | **<0.0001** | 0.0064 | 20.67 | **<0.0001** | 0.0048 | 13.81 | **<0.0001** |
| SFP- LIP | 0.0015 | 6.27 | **<0.0001** | 0.0016 | 7.17 | **<0.0001** | 0.0013 | 4.751 | **<0.0001** |
| SFP-PD | 0.0020 | 6.08 | **<0.0001** | 0.0038 | 12.15 | **<0.0001** | 0.0031 | 8.64 | **<0.0001** |
| LIP-PD | 0.0004 | 1.33 | 0.544 | 0.0022 | 7.02 | **<0.0001** | 0.0017 | 4.9 | **<0.0001** |
