# Supplementary material for: Different dry-wet pulses favor different functional strategies: A test using tropical dry forest tree species
Source: PLoS One. 2024 Dec 3;19(12):e0309510. doi: 10.1371/journal.pone.0309510 (PMC11614228; doi:10.1371/journal.pone.0309510)
Supplement: S6 Table — (DOCX) [file pone.0309510.s009.docx]

S9 Table. Effects of species functional strategy (PC1 or PC2 scores) and dry-wet pulse scenario on survival, while controlling by plant height, for saplings of 18 TDF species in a greenhouse experiment. (A) generalized linear mixed model for survival against PC1 (R^2^_m_ = 0.24; R^2^_c_ = 0.78) and B) generalized linear mixed model for survival against PC2 (R^2^_m_ = 0.4; R^2^_c_ = 0.81). X^2^ values correspond to Wald Type III test statistics.

| Continuum of  functional strategies | Predictors | *X^2^* | DF | Pr(>Chisq) |
| --- | --- | --- | --- | --- |
| 1. PC1   (avoidance-  tolerance) | Height | 0.2666 | 1 | 0.6056 |
|  | PC1 | 0.0001 | 1 | 0.9919 |
|  | Dry-wet pulse | 0.0001 | 1 | 0.9918 |
|  | PC1*Dry-wet pulse scenario | 0.0001 | 1 | 0.9909 |
| 1. PC2   (conservative-acquisitive) | Height | 0.2774 | 1 | 0.5984 |
|  | PC2 | 0.0371 | 1 | 0.8472 |
|  | Dry-wet pulse | 0.0006 | 1 | 0.9810 |
|  | PC2*Dry-wet-pulse scenario | 0.0002 | 1 | 0.9879 |
