# Supplementary material for: Different dry-wet pulses favor different functional strategies: A test using tropical dry forest tree species
Source: PLoS One. 2024 Dec 3;19(12):e0309510. doi: 10.1371/journal.pone.0309510 (PMC11614228; doi:10.1371/journal.pone.0309510)
Supplement: S7 Table — (DOCX) [file pone.0309510.s010.docx]

S10 Table.- Effects of species functional strategy (PC1 or PC2 scores), while controlling for initial height, on survival after individuals suffered 30% dieback under greenhouse conditions. A) generalized linear mixed model for PC1 (R^2^_m =_ 0.19_;_ R^2^c=0.74) and B) generalized lineal model for PC2 (R^2^_m =_ 0.04_;_ R^2^c=0.71). Wald Type III test statistics are shown.

|  | **Predictors** | **Df** | ***X^2^*** | ***P*** |  |
| --- | --- | --- | --- | --- | --- |
| **A)** **PC1** | Height | 1 | 4.8877 | <0.05 |  |
|  | PC1 | 1 | 4.6127 | **<0.05** |  |
| **B)** **PC2** | Height | 1 | 4.7556 | <0.05 |  |
|  | PC2 | 1 | 0.2082 | 0.6482 |  |
